# The Current Status of Allogenic Islet Cell Transplantation

**DOI:** 10.3390/cells12202423

**Published:** 2023-10-10

**Authors:** Zofia Czarnecka, Nidheesh Dadheech, Haide Razavy, Rena Pawlick, A. M. James Shapiro

**Affiliations:** Department of Surgery, University of Alberta, Edmonton, AB T6G 2RW3, Canada; dadheech@ualberta.ca (N.D.); hrazavy@ualberta.ca (H.R.); rpawlick@ualberta.ca (R.P.); jshapiro@ualberta.ca (A.M.J.S.)

**Keywords:** type 1 diabetes, islet, allogenic, transplantation, stem cell, immunosuppression, iPSC

## Abstract

Type 1 Diabetes (T1D) is an autoimmune destruction of pancreatic beta cells. The development of the Edmonton Protocol for islet transplantation in 2000 revolutionized T1D treatment and offered a glimpse at a cure for the disease. In 2022, the 20-year follow-up findings of islet cell transplantation demonstrated the long-term safety of islet cell transplantation despite chronic immunosuppression. The Edmonton Protocol, however, remains limited by two obstacles: scarce organ donor availability and risks associated with chronic immunosuppression. To overcome these challenges, the search has begun for an alternative cell source. In 2006, pluripotency genomic factors, coined “Yamanaka Factors,” were discovered, which reprogram mature somatic cells back to their embryonic, pluripotent form (iPSC). iPSCs can then be differentiated into specialized cell types, including islet cells. This discovery has opened a gateway to a personalized medicine approach to treating diabetes, circumventing the issues of donor supply and immunosuppression. In this review, we present a brief history of allogenic islet cell transplantation from the early days of pancreatic remnant transplantation to present work on encapsulating stem cell-derived cells. We review data on long-term outcomes and the ongoing challenges of allogenic islet cell and stem cell-derived islet cell transplant.

## 1. Introduction

Type 1 Diabetes (T1D) is an autoimmune destruction of pancreatic beta cells. In 2022, there were approximately 8.7 million cases of T1D worldwide, with the prevalence continuing to increase substantially [1,2]. It is projected that by 2040, there will be 17.4 million cases, which will pose an escalating financial burden for healthcare systems [2]. In Canada, it was recently estimated that 30 billion dollars are spent managing all forms of diabetes and its complications, and these costs are rapidly becoming non-sustainable [3]. While there have been exciting technological advances in continuous glucose monitors and less invasive insulin pump delivery systems, the burden of T1D remains vast. Poorly regulated and fluctuating glucose levels can lead to severe complications for patients, including renal dysfunction, vascular disease, blindness and early death [4].

**Figure 1:**
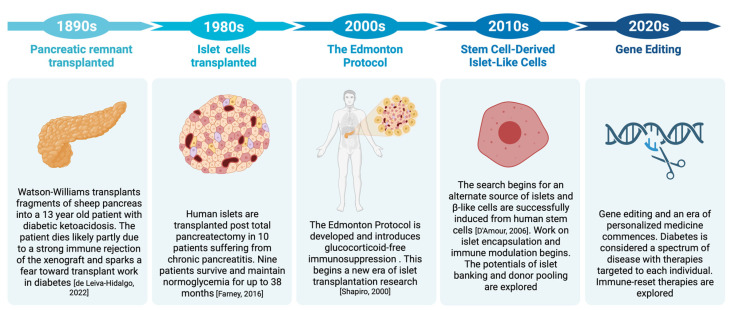
The evolution of allogenic islet cell transplantation highlighting the main landmarks and directions of research from 1890 to the present day [5,6,7,8].

The development of an islet transplantation protocol in 2000 by Shapiro et al. revolutionized T1D treatment and offered a glimpse at a cure for the disease. Since then, advances have been made in optimizing islet cell transplantation as well as exploring alternative sources of islets, notably stem cell-derived islet cells and different transplant sites. In this review, we present a brief history of allogenic islet cell transplantation from the early days of transplanting pancreatic remnants to present-day work on encapsulating stem cell-derived cells (Figure 1). We also review recent data on long-term outcomes and the ongoing challenges of allogenic islet cell transplant.

## 2. A Brief History of Cell Transplantation for the Treatment of Diabetes

**Figure 2:**
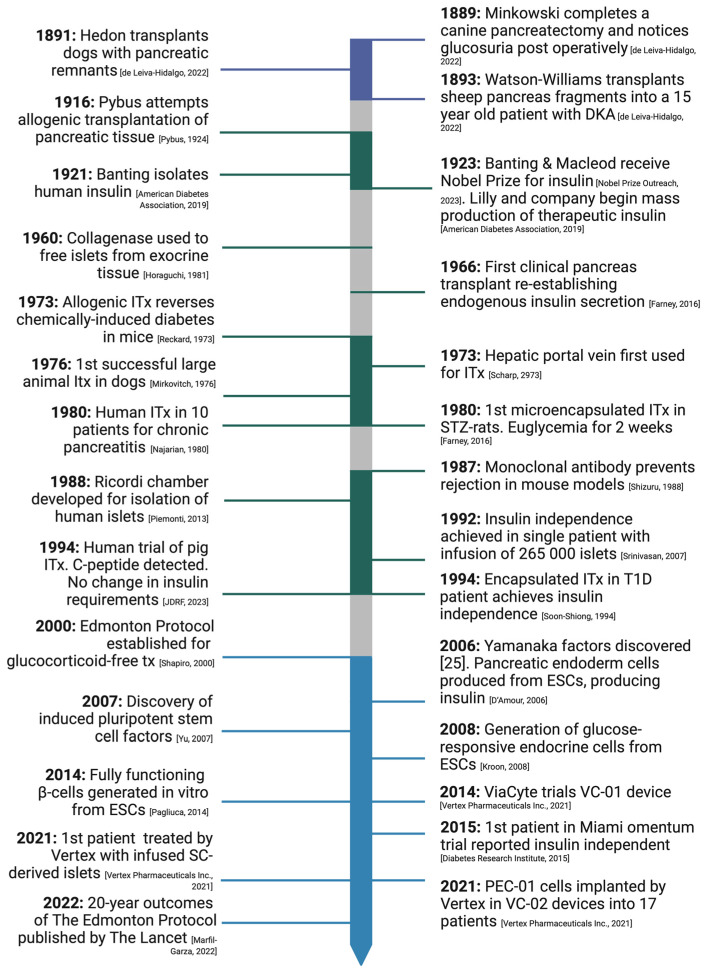
The history of islet cell transplantation (ITx) and landmark discoveries from 1889 to 2021. DKA = diabetic ketoacidosis; ITx = islet cell transplantation; Tx = transplantation; ESC = embryonic stem cell [5,6,7,8,9,10,11,12,13,14,15,16,17,18,19,20,21,22,23,24,25,26,27].

The origin of transplantation as a treatment for diabetes dates back to the 1800s (Figure 2), before islet cells had been isolated and properly understood. Oskar Minkowski is credited with the initial discovery of the pancreatic origin of diabetes after conducting total pancreatectomies in dogs in 1889 and noticing the development of glucosuria immediately postoperatively [5]. Two years later, Emmanuel Hedon furthered Minkowski’s research by auto-transplanting pancreatic remnants subcutaneously into de-pancreatized dogs. His experiments illustrated that if the transplanted remnant remained in place with an adequate vascular supply, the dog would survive. However, as soon as the graft was removed, there was a rapid onset of glycosuria and progression to death [5]. In 1893, Watson-Williams attempted the first pancreatic transplant in humans by transplanting pieces of sheep’s pancreas subcutaneously into a 13-year-old patient with diabetic ketoacidosis [5]. The xenograft experiment failed, and the patient died a few days later, triggering a turn away from focusing on the pancreatic role in diabetes.

The next breakthrough came in the early 1900s with experiments involving pancreatic duct ligation and intraperitoneal injection of “extracts of degenerated pancreas” by Eugene Gley [5]. He published his findings in 1900, summarizing the metabolic effects of his pancreatic extract. In 1916, Frederick Pybus attempted the first allogenic transplantation of pancreatic tissue. He grafted slices of pancreatic tissue subcutaneously into the abdominal walls of two pancreatic patients. In one patient, a brief improvement in glycosuria was noted [9]. In 1920, Banting elaborated on these experiments by ligating the pancreatic duct in dogs and demonstrating the resulting acinar degeneration and recovery of internal secretions [10]. In 1922, exogenous insulin was isolated. Shortly after the discovery of insulin earned Banting and Macleod the 1923 Nobel Prize in Medicine [11], Lilly and the company began mass production and distribution of therapeutic insulin [10].

Although the early 1900s improved the understanding of islet cells and their role in insulin secretion, islets were challenging to isolate. In 1960, Horaguchi and Merrell introduced the technique of perfusing the pancreas with collagenase to detach islets from the collagen matrix [12]; this sparked a new era of discovery. In 1973, Reckard and Barker were the first to use islet transplantation to reverse diabetes in a chemically induced mouse model [13]. The following year, the hepatic portal vein was used as a site for islet transplantation in rodents [14]. In 1980, the first human islet cell transplant was carried out in 10 patients post pancreatectomy for chronic pancreatitis. Nine of these patients survived and maintained normoglycemia for up to 38 months [16]. However, early islet cell transplantation protocols required high doses of steroids for immunosuppression, contributing to islet cell toxicity as well as marked patient side effects.

In 2000, Shapiro et al. developed the Edmonton Protocol for islet cell transplantation [7] and introduced a glucocorticoid-free immunosuppression regime. They also described the required islet cell equivalent needed for successful transplantation and maintenance of euglycemia; this illustrated the first approach to a cell-based cure for diabetes.

## 3. Islet Transplantation: Long-Term Outcomes and Recent Updates

In 2006, Shapiro et al. published their follow-up findings of 36 patients treated with pancreatic islet cell transplantation at nine international sites [28]. Per the Edmonton Protocol, islets were isolated from deceased donors and then infused into the recipient’s portal vein. Of the participating patients, 58% were insulin-independent with adequate glycemic control at some point during the study. A total of 76% of these patients required some level of insulin at two years after transplant [28]. Importantly, this study illustrated the damaging effect of immunosuppressive drugs on islet cell function and prompted subsequent research into single-agent immunosuppression. Immunosuppressive drugs are particularly toxic in the portal hepatic site [29], and this has led to further investigations of alternate transplantation sites for islets.

In 2022, Marfil-Garza et al. published the 20-year findings of pancreatic islet cell transplantation from the University of Alberta in Edmonton, Canada [27]; this was the largest cohort study of long-term outcomes following islet transplantation, including 255 patients from Edmonton alone, and illustrated the long-term safety of islet cell transplantation despite chronic immunosuppression. Over the median follow-up of 7.4 years, 90% of patients survived with a median graft survival time of 5.9 years. Those patients with sustained graft survival demonstrated better insulin independence as well as better sustained glycemic control compared with patients with non-sustained graft survival. The largest independent predictor of sustained graft survival in this study was the peri transplant use of dual anti-inflammatory therapies: anakinra plus etanercept [27].

These findings are consistent with the data published by the Collaborative Islet Transplant Registry (CITR) in their 5-year outcome data of islet transplantation in 398 patients with T1D complicated by severe hypoglycemia events [30]. The CITR study further described four predictive factors of sustained graft survival following islet transplantation: recipient age ≥35 years, ≥325,000 total infused islet equivalents, immunosuppression with T cell depletion or TNF-a inhibition, and post-transplant use of mTOR and calcineurin inhibitors. Of the patients meeting these four criteria, 53% were insulin independent at five years following transplant, 76% had an HbA1c under 7.0%, and 95% were free of severe hypoglycemic events. Of note, the absence of severe hypoglycemia events was similar in all age groups when the remaining three criteria were present, and the authors emphasized that age over 35 should, therefore, not preclude patients from receiving islet cell transplantation [30].

The largest retrospective, multicenter cohort study from the CITR was recently published by The Lancet, presenting the five-year outcome data for 1210 patients who received islet transplantation or islet-after-kidney transplantation in the last 20 years [31]. The primary conclusion of this study was that early primary graft function is an independent predictor of long-term islet transplantation outcomes [31].

In this study, islet graft function was measured 28 days following the last islet infusion and calculated as the BETA-2 score using fasting C-peptide and glucose levels and exogenous insulin needs; this revealed a linear relationship between primary graft function and long-term transplant success, which was independent of patient factors and immunosuppressive strategies. The mean primary graft function of patients was quantified using the BETA-2 score and was inversely related to the cumulative 5-year incidence of unsuccessful islet transplant of 70.7% [31]. What remains to be assessed prospectively is whether primary graft function can be used to plan and time additional islet cell infusions.

Overall, these recent studies and clinical data results support the safety and efficacy of islet cell transplantation for the treatment of T1D and provide promising new directions for future research in optimizing the long-term success of transplanted islets.

## 4. Stem Cells: A New Cell Supply

While the advances made in improving the Edmonton Protocol are promising, they remain limited by two main obstacles: scarce organ donor availability and risks associated with chronic immunosuppression. To overcome these challenges, the search has begun for an alternative cell source. The most promising avenue is the exploration of stem cell-derived islet-like cells. Islets can be derived from embryonic stem cells (ESC) or induced from pluripotent stem cells (iPSC).

The derivation of islets from ESCs has been successfully carried out since the early 2000s. In 2006, D’Amour et al. generated pancreatic endoderm cells from ESCs, which expressed insulin at a similar level to human islets but were not yet responsive to glucose stimulation [8]. In 2008, Kroon et al. followed this with the generation of glucose-responsive endocrine cells generated from human ESCs. They successfully transplanted these cells into diabetic mice and showed that over several months, these cells differentiated into functional ß-cells and maintained euglycemia by secreting insulin in response to glucose levels [23]. Then, in 2014, Pagliuca et al. successfully generated fully functioning pancreatic ß-cells in vitro from ESCs [24]. This differentiation protocol has formed the foundation of human clinical trials carried out by two companies: ViaCyte and Vertex [32].

In 2022, Vertex announced in public media the results of their first trial patient with T1D who received an intraportal infusion of ESC-derived islet cells. They showed an increase in both fasting and post-prandial C-peptide levels, and the first patient attained complete insulin independence [25]. These trials required chronic high-dose immunosuppression to prevent allogeneic rejection of the cells.

ViaCyte, on the other hand, encapsulated ESC-derived pancreatic endoderm cells in a biologic membrane device, which was then implanted subcutaneously. VC-01, ViaCyte’s first cell-based therapy, encapsulated pancreatic progenitor cells (PEC-01) derived from ESCs in a device called PEC-Encap. These cells further matured into ß-like cells once implanted subcutaneously. ViaCyte then improved this therapy in VC-02, where they used the same implant model but with more mature and functional cells (PEC-02). The preliminary results from 17 patients with T1D who received this transplant were shared in 2021. Six of the 17 patients had detectable C-peptide secretion six months post-transplant [33]. Both clinical trials, although early, demonstrate the potential for ESCs to create a renewable source of islet cells. ViaCyte Inc. was recently acquired by Vertex Inc.

While ESC-derived islet cells address the problem of islet and donor supply, recipients still require chronic immunosuppression. In response, a new direction of study has emerged with the use of pluripotent stem cells. In 2006, Takahashi and Yamanaka described the use of pluripotency genomic factors, coined “Yamanaka factors,” which can reprogram mature somatic cells back to their embryonic, pluripotent form—known as inducible pluripotent stem cells (iPSCs) [34]. iPSCs can then be cultured and differentiated into specialized cell types, including ß -like islet cells. This discovery has opened a gateway to a personalized medicine approach to treating diabetes, which also circumvents the issues of donor supply and immunosuppression. Under this model, a patient with diabetes could have their blood cells transformed into iPSCs and then differentiated into ß-like islet cells, which could then be transplanted therapeutically as a cell-based cure for diabetes without the need for immunosuppression. The Edmonton Protocol for islet cell transplant already serves as a proof of concept for this model, and clinical trials with ESCs have demonstrated the potential for stem cells to serve as an alternate source of islets. The use of iPSCs would incorporate lessons learned from these clinical trials and further accelerate the work toward a sustainable cure for diabetes.

**Figure 3:**
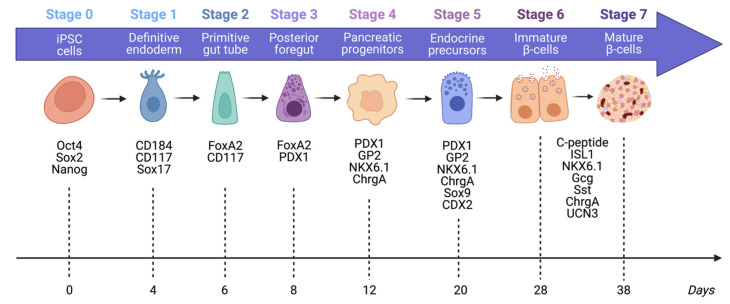
The seven stages of differentiation of iPSCs into mature ß-like cells, as well as the time taken to reach each stage in a number of days. At each stage, cells express unique markers, which can be tested to assess the quality of differentiation.

iPSCs differentiate and mature into islet-like cells through a seven-stage process, as illustrated in Figure 3. The final extended maturation at the end of this process was recently optimized by Balboa et al. [35]. By altering the composition of the proliferation culture medium, the proportion of insulin-positive cells remained stable while the number of off-target cells and polyhormonal cells expressing both insulin and glucagon decreased. They also further matured the beta cells in vitro and showed that the cells were able to acquire biphasic glucose-stimulated insulin secretion and undergo cytoarchitecture reorganization and maturation of signalling transduction pathways that mimicked human islet cells. Although complete transcriptome modulation and maturation occur in vivo, when these cells were transplanted into mouse models, the more mature stem cell islets demonstrated higher C-peptide secretion and better glucose regulation compared to more immature stem cell islets [35]. Further work is underway to optimize the extended maturation (“seventh stage”) of stem cell islets.

Another recent advancement in optimizing iPSC maturation is the improved three-dimensional cell expansion protocol. Cuesta-Gomez et al. (2023) demonstrated that 3D expanded cells showed higher expression of pluripotency markers (Oct4, Nanog, Sox2) compared with cells cultured in 2D planar models (69.4% vs. 57.4%) [36]. Traditionally, these iPSC cell lines have demonstrated increased mutations at high passage numbers, and PCR analysis of the 3D cultured iPSCs demonstrated no mutations at the eight most commonly susceptible regions. Finally, when transplanted into mouse models, the 3D expanded cells produced more cystic teratomas, while the 2D planar cells produced more solid teratomas. As a result, they concluded that 3D expansion of iPSCs allows for 100 times higher expansion potential than in 2D plates, and the cells produced from 3D expansion in bioreactors demonstrate enhanced pluripotency and potentially less off-target growth than the 2D cells [36].

Optimizing stem cell-derived islet cells is rapidly progressing. These cells remain a promising alternative to islet cells and have the potential to have the same efficacy as human islet cells. Further work is needed to limit off-target growth and cyst formation and to determine the best site for implantation of these cells therapeutically.

## 5. Ongoing Challenges of Allogenic Islet and Stem Cell Transplantation

**Figure 4:**
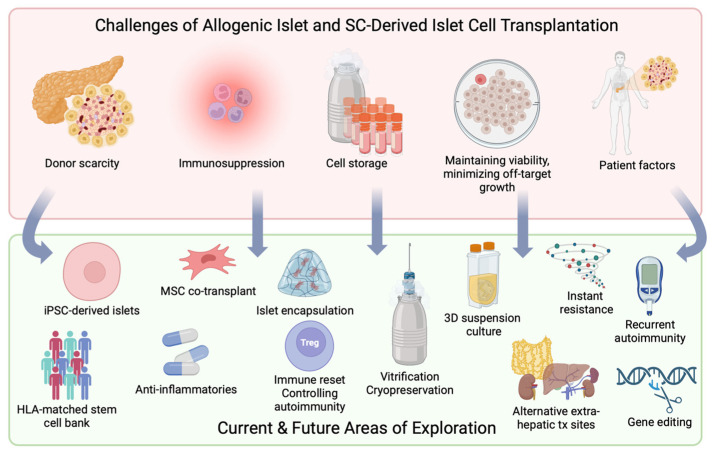
Ongoing challenges to allogenic and stem cell (SC) derived islet transplantation and the current areas of exploration for solutions to these obstacles.

Several challenges remain with allogenic islet cell transplantation, as summarized in Figure 4. One of the greatest barriers is the required use of chronic immunosuppression to prevent islet cell rejection. The concerns with immunosuppression are twofold: patient and graft adverse effects. From a patient perspective, systemic immunosuppression exposes patients to opportunistic infections following transplant, increased risk of insulin resistance, as well as the potential of neoplasm formation. From a graft perspective, immunosuppression regimens are associated with islet graft failure and cell apoptosis. Novel approaches to overcoming the challenges of immunosuppression have been explored with the use of anti-inflammatories, immunomodulatory therapies, and the co-transplantation of islet cells with accessory cells for local immunosuppression.

### 5.1. Immunosuppression

Anti-inflammatories have been shown to improve islet cell survival post-transplant and minimize inflammatory responses after infusion. Two agents that are used are etanercept (TNFα blocker) and anakinra (IL-1 receptor antagonist). Etanercept neutralizes lymphotoxins while anakinra prevents ß-cell destruction and minimizes the instant blood-mediated inflammatory reaction that occurs after islet cell transplantation [37]. In their 2011 study, McCall et al. demonstrated improved islet engraftment when these two agents are used in combination. Together, islet engraftment improved to 87.5% compared with 45.5% with etanercept alone or 53.9% with anakinra alone [38]. This results in improved insulin production and reduced beta-cell apoptosis and is being used in the peri-transplant period to mitigate innate inflammatory responses [38]. Additionally, when anakinra and etanercept were used in combination as dual therapy, there was no increase in overall infection risk, as assessed by Szempruch et al. in their 2019 systematic review [37]. Another promising agent in this field is alpha-1-antitrypsin, a serine protease inhibitor, which inhibits interferon-gamma-induced macrophage activation, thereby improving islet cell engraftment by minimizing cytokine-induced inflammation [39].

Once islet cells are transplanted, and the initial inflammatory response is overcome, the next barrier to graft survival is graft acceptance and tolerance by the host. One novel approach is the co-infusion of islet cells with regulatory T cells (Tregs). Tregs are immunomodulatory cells that play a role in preventing autoimmune rejection. A 2016 study by Visperas and Vignali investigated the role of Tregs in causing T1D [40]. They argued that a principal cause of the development of T1D is the failure to maintain stability and survival of Tregs in response to genetic and environmental factors [40]; this has prompted an investigation into boosting Tregs, inhibiting destabilizing pathways, or boosting the number of Treg cells in transplant recipients to promote the survival of islet grafts. In a two-year study conducted by Zielinski et al., they treated pediatric T1D patients with a combined infusion of Tregs and rituximab and demonstrated delayed progression of disease compared with monotherapy of Tregs or rituximab alone [41]. Patients receiving the combined therapy maintained higher stimulated and fasting C-peptide levels compared with the monotherapy and control groups. Patients receiving Tregs alone also demonstrated higher C-peptide levels than the untreated control patients [41]. While this study was small, with only 11–13 patients in each cohort, the results illustrate the potential transplant tolerance benefits from Tregs treatment. A human clinical trial (NCT03182426) is presently underway at our site in Edmonton, Canada, looking at the benefit of T-cell depletion combined with the benefits of dual anti-inflammatory therapy, as detailed in the previous section, to create an “immunologic reset” [42]. Patients with T1D receive Plerixafor in stage one (causing systemic mobilization of CD34+ stem cells for ß-cell repair). Then, in stage two, they receive alemtuzumab, anakinra, etanercept and liraglutide to deplete T-cells, mitigate the anti-inflammatory response and stimulate ß-cell regeneration; this is being tested in patients with new-onset T1D to assess whether this will preserve endogenous islet cell function and insulin secretion [42]. If successful, this approach may be beneficial in islet cell transplant recipients as a means of preventing rejection without the need for chronic immunosuppression.

Finally, another approach to mitigating the challenge of immunosuppression is the co-transplantation of islets with mesenchymal stromal cells (MSCs) as accessory cells. Wang et al. (2022) engineered MSCs to overexpress PD-L1 and CTLA4-Ig [43]. In vitro, this resulted in suppressed activation of CD4+ and CD8+ T cells. They further proved the efficacy of this model in allogenic mouse transplant models, where MSCs were co-transplanted with islets into mice kidney capsules; this allowed for functioning islets for 100 days without any systemic immunosuppression. Comparatively, islets transplanted in the contralateral kidney without MSCs were promptly rejected, and mice transplanted with islets without any MSCs at all rejected islets within two weeks of transplant [43]. In a cynomolgus monkey model, Kenyon et al. (2021) also demonstrated the immunomodulatory effects of MSCs co-transplanted with islet cells [44]. They were able to prolong the anti-rejection period by infusing transplant-recipient monkeys with intravenous recipient MSCs for a month after transplantation [44].

MSC co-transplantation has also been shown to improve the vascularization of the transplanted islet graft in a mouse model by promoting endothelial and smooth muscle cell proliferation and migration, further improving graft survival [45]. In another cynomolgus monkey experiment of co-transplantation with MSCs by Berman et al. (2010), improved vascularization and islet engraftment was demonstrated in the co-transplantation group compared to the group transplanted with only islets [46].

These experiments demonstrate local immunomodulation without the need for any physical barriers between the islets and host or any manner of cell encapsulation. While promising, further investigations are needed to explore the long-term efficacy of this approach, whether it can be translated to other transplant sites such as the portal vein or subcutaneous space, and whether the same degree of immunosuppression and islet survival can be achieved with allogeneic MSCs.

### 5.2. Encapsulation

The immediate blood-mediated inflammatory reaction discussed above is incredibly detrimental to islet cells. Mitigating this has been challenging as islet cells are more prone to hypoxia than other cells and require high levels of oxygen to engraft and survive [47]. By encapsulating islets in a biological barrier, the host’s immune cells can be kept away from the islets while still allowing the diffusion of insulin, oxygen, and nutrients.

Encapsulation can be broadly divided into two categories: micro and macro-encapsulation. Micro-encapsulation techniques involve coating islet cells in a thin natural polymer, traditionally consisting of alginate. Alginate hydrogels are modifiable in firmness and permeability. When mixed with islet cells and then injected into the recipient, the hydrogel polymerizes into a fibrin gel, creating a protective porous nest for the islets [48]. Different combinations of alginate-containing hydrogels have been tested with normoglycemia achieved in diabetic mouse and pig models for one month [49].

Some of the challenges encountered with microencapsulation are the inflammatory foreign body response to the polymer as well as the injectable nature of the mixture; this does not allow for retrieval of the graft if problems later arise. These questions prompted the development of macro-encapsulation techniques, which involve implanting devices over 1 mm in size [48]; this allows for a scaffold or pouch that can be easily implanted, monitored, and removed if needed. ViaCyte Inc. is presently testing a microencapsulation device which contains a plastic support scaffold externally with a thin mesh-like surface internally for islets [48]. Their 2017 trial of their device showed good oxygen diffusion and successful albeit low-level insulin secretion in patients with diabetes, confirming that islets maintain their endocrine function with microencapsulation [48]. Presently, biotechnology companies are working to minimize islet cell clumping within their devices to maintain equal and adequate oxygen delivery throughout the devices.

### 5.3. Vitrification of Islet Cells

In 2022, Zhan et al. introduced the potential for “islet banking” by developing a novel vitrification process for islet cells [50]. Traditionally, islets cannot be preserved more than 48–72 h post isolation, and studies have shown that the quality of islets degrades the longer the time between isolation and transplantation. Prior to this study, other centers had attempted vitrification but only achieved an average of 55–62% islet viability after thawing. Zhan et al. theorized that this poor viability was due to excessive volume of cryoprotectant and low islet density, resulting in a slow speed of cooling and warming [50].

Their group instead proposed suspending the islets in cryoprotectant within a nylon mesh, which wicks excess cryoprotectant from the cells, increasing islet density prior to submersion in liquid nitrogen; this increased the rate of cooling and warming. They subsequently described the optimal composition of cryoprotectant for islets using a mixture of ethylene glycol and dimethyl sulfoxide to maintain islet viability. Their methods achieved over 90% of islet cell recovery and viability, with cells showing similar morphology, function and insulin secretion when compared to non-vitrified islets [50].

The results of Zhan et al. lay a foundation for further experimental directions, including testing this protocol in different batches of stem cell-derived islet cells to assess for variability in cell function post vitrification as well as evaluation of this technique to assess for transportation of cells between centers.

### 5.4. Optimal Transplant Site

Traditionally, islet cells have been transplanted into the hepatic portal vein as the only successful routine clinical site [7]. This minimally invasive approach using percutaneous ultrasound and fluoroscopic-guided access allows for oxygenation and rapid perfusion of islet cells. Due to a combination of factors, there is a large loss of islet cells, often prompting two or three islet cell infusions per recipient to maintain adequate insulin secretion. As more understanding emerges around the innate inflammatory response and immune modulation following islet cell infusion, different organs and sites are being explored to identify the best location for islet cell transplant; this is particularly of interest as research shifts towards stem cell-derived islet-like cells, and the need for an easily accessible transplant and retrieval site becomes a priority. Two promising areas are the subcutaneous space and the omentum.

The subcutaneous space is relatively avascular; however, it is easily accessible and lends itself well to the introduction of biomaterial or macro-encapsulated islet cells. These bioscaffolds mimic an extracellular matrix and are based on natural polymers such as collagen, fibrin, and alginate [48]. Their design promotes angiogenesis in the area, prompting a two-phase procedure: first, the implant of the biomaterial to form a well-vascularized cavity. And then secondly, the incorporation of islet cells; this was demonstrated by Pepper et al. in 2015 with the temporary placement of a vascular access catheter in the subcutaneous space in mice with streptozotocin-induced diabetes [51]. This catheter was used to prime the area for four weeks and then removed, and islet cells were introduced into the newly vascularized cavity. Diabetes was successfully reduced in 91% of mice, and normoglycemia was maintained for over 100 days [51].

More recently, Yu et al. demonstrated successful device-less islet transplantation subcutaneously without the need for priming by incorporating islets in an “islet viability matrix” composed of collagen, glutamine, fetal bovine serum and medium [52]. They demonstrated sustained euglycemia in both immune-competent and incompetent murine and porcine models using murine, human, and porcine islet cells [52]. While this study did not address the challenge of immunosuppression, it offered promising insight into the use of the subcutaneous space for islet cell transplantation.

Another potential site for islet cell transplant is the omentum. Its large surface area, high vascularity and portal venous drainage system make it an opportune transplant site [53]. A clinical trial underway in Miami (NCT02213003) involves transplanting islets into patients with T1D onto the omentum [54]; this is carried out laparoscopically, and the islets are mixed with human thrombin to create a biological mesh that adheres to the omentum. A preliminary case report was published in 2017 from this study. It reported the case of a 43-year-old female patient with a 25-year history of diabetes who had 600,000 islet equivalents laparoscopically transplanted onto her omentum. She was able to discontinue her insulin just 17 days after the transplant. She maintained insulin independence with stable glycemic control 12 months after her procedure, although she was noted to have decreased insulin secretion and increased glucose levels [55]. Further follow-up has not yet been reported. In collaboration with the University of Miami, a partner study is ongoing at the University of Alberta (NCT02821026) with the same method of omental transplantation and immunosuppression [56]. Recruitment is ongoing at this site as well but with only more limited success to date.

While the laparoscopic approach is promising for omental transplants, one limitation of this approach is the difficulty of subsequent islet cell transplants due to adhesions, risks of wound infections and impaired healing with repeat invasive surgeries, particularly if converted to a laparotomy [53]. Nevertheless, the omentum remains a promising potential site.

## 6. Conclusions

Allogenic islet cell transplantation has evolved considerably since the early experiments of Oscar Minkowski, Emmanuel Hedon, and Eugene Gley. The Edmonton Protocol of human islet cell transplantation was built on the foundation of knowledge developed by these pioneers. Recent advances have optimized the long-term success of transplanted islet cells, and in parallel, stem cell technology has introduced the possibility of an alternate source of islets.

As researchers around the world race to develop the final cure for diabetes, new developments and discoveries are rapidly emerging. This review is a testament to the vast amount of knowledge and information that has been discovered in the last few years alone and the exciting new avenues for further exploration.

## Data Availability

No new data were created or analyzed in this study. Data sharing is not applicable to this article.
